# Consequences of Patellar Tendinopathy on Isokinetic Knee Strength and Jumps in Professional Volleyball Players

**DOI:** 10.3390/s22093590

**Published:** 2022-05-09

**Authors:** Marie Chantrelle, Pierre Menu, Marie Gernigon, Bastien Louguet, Marc Dauty, Alban Fouasson-Chailloux

**Affiliations:** 1 Service de Médecine Physique et Réadaptation Locomotrice et Respiratoire, CHU Nantes, Nantes Université, 44093 Nantes, France; marie.chantrelle@chu-nantes.fr (M.C.); pierre.menu@chu-nantes.fr (P.M.); marc.dauty@chu-nantes.fr (M.D.); 2 Département de Médecine Physique et de Réadaptation, Centre Hospitalier Universitaire d’Angers, 49000 Angers, France; 3 Service de Médecine du Sport, CHU Nantes, Nantes Université, 44093 Nantes, France; bastien.louguet@chu-nantes.fr; 4 Institut Régional de Médecine du Sport (IRMS), 44093 Nantes, France; 5 Inserm, UMR 1229, RMeS, Regenerative Medicine and Skeleton, ONIRIS, Nantes Université, 44042 Nantes, France; 6 CIAMS, Complexité, Innovation, Activités Motrices et Sportives, Université Paris-Saclay, 91405 Orsay, France; marie.gernigon@universite-paris-saclay.fr; 7 CIAMS, Complexité, Innovation, Activités Motrices et Sportives, Université d’Orléans, 45067 Orléans, France

**Keywords:** patellar tendinopathy, volleyball, jumps, strength, VISA-P score

## Abstract

Patellar tendinopathy (PT) in professional volleyball players can have an impact on their careers. We evaluated the impact of this pathology in this specific population in terms of isokinetic strength and jumping performances. Thirty-six professional male volleyball players (mean age: 24.8 ± 5.2) performed isokinetic knee assessments, single-leg countermovement jumps and one leg hop test. They filled out the Victorian Institute of Sport Assessment-Patella (VISA-P) score. Two groups were assessed: “PT group” (*n* = 15) and “control group” (*n* = 21). The VISA-P score was lower in the PT group (*p* < 0.0001). No difference was found between the isokinetic strength limb symmetry index and the jump performance limb symmetry index. The healthy legs of the control group were compared with the affected (PT+) and the unaffected legs (PT−) of the PT group. Compared with the healthy legs, both PT+ and PT− legs showed decreased values of quadriceps and hamstring strengths. Only PT+ legs scored lower than healthy legs in countermovement jumps and hop tests. No differences were found between PT+ and PT− legs for muscle strengths and jumps. A low correlation existed between quadriceps strength and jumping performances (*r* > 0.3; *p* < 0.001). Volleyball players with PT showed a decrease in the isokinetic knee strength. This strength deficit was found both on the symptomatic legs and the asymptomatic ones. Jumps were only significantly altered on the pathological legs. Highlighting that the unaffected limbs were also impaired in addition to the affected limbs may help provide a better adaptation of the rehabilitation management.

## 1. Introduction

Patellar tendinopathy (PT), commonly called « Jumper’s Knee », is an overuse injury resulting in the presence of small tears in the knee extensor system [1]. This painful condition is usually due to sports such as basketball or volleyball because of high quadriceps loadings secondary to repetitive jumps [2]. This clinical diagnosis has no gold standard in terms of imaging [3]. In all types of elite athletes, the prevalence of PT is estimated at about 14%, whereas in male volleyball players, it is about 45% [4]. In female volleyball players, the prevalence seems lower, around 20% [5]. The ultimate consequence of this common disease for these athletes is the risk of a premature career-ending [4]. Indeed, up to 50% of symptomatic players have to quit their sports careers because of knee pains [6]. In the best cases, return-to-sport could be delayed from a few days to several weeks [7,8].

Thus, the prevention and the identification of PT are essential. Many studies examined risk factors, but the results remain inconsistent. Indeed, in 2011, a study reported nine factors associated with PT: weight, body mass index (BMI), waist-to-hip ratio, leg-length difference, the arch height of the foot, quadriceps and hamstring flexibilities, quadriceps strength and vertical jump performance [9]. However, in 2020, another study reported no intrinsic risk factors but found that vertical jumping ability, ankle dorsiflexion range of motion, dynamic balance, dynamic knee alignment and landing mechanics were associated with the development of a PT [10]. Other factors such as gender, height and weight remain debatable [11]. Moreover, the volume of activity (jump training, number of volleyball sets played per week, Countermovement Jump (CMJ) height) seems to be a modifiable risk factor for PT [12]. Therefore, it is still difficult to determine the real risk of developing PT for athletes.

Currently, no difference in knee extensor strength was shown between athletes with and without PT in the prospective assessment [13]. Nevertheless, Crossley et al. [14] reported a decreased knee extensor strength but also a decreased jumping performance. Thus, jump evaluation and isokinetic testing seem to be appropriate in this perspective [15]. Indeed, PT is a diagnosis based on physical examination and typical patient history [16], tools reflecting the impairment associated with PT are relevant for its evaluation. An early and accurate assessment may therefore allow personalized management of the PT in order to limit time-loss [17].

The main objective of this study was to evaluate the isokinetic knee strength and jump performances of professional volleyball players, with PT and without PT, at the beginning of three sports seasons. The secondary aim was to assess the relationship between knee strength and jump ability. Indeed, we looked for a correlation between isokinetic knee strength and jumping performances in order to assess the functional impact of a PT.

## 2. Materials and Methods

### 2.1. Participants and Recruitment

A professional team of male volleyball players was included during the systematic pre-season evaluation at the start of three sports seasons from 2017 to 2020 in the Sports Medicine department of the University Hospital of Nantes, France.

Players of the PT group had to present a typical history of pain and consistent clinical findings such as tenderness on palpation of the patellar tendon [2,16,18,19]. No ultrasound or magnetic resonance imaging was needed because of the lack of a gold standard for the use of morphologic exams to diagnose PT [3]. Players of the control group had to be free of any pain in both lower limbs (“healthy legs”). In the PT group, we distinguished the symptomatic legs (“PT+ legs”) and the asymptomatic ones (“PT− legs”). To be included, all volleyball players had to be able to train without limitations. In case of cessation of training, previous knee surgery or bilateral PT, the volleyball player was excluded.

The pre-season evaluation consisted of measuring the isokinetic knee strength and performing single-leg CMJ and one-leg hop tests in random order. Moreover, the volleyball players’ symptoms and their sportive activities were assessed by the Victorian Institute of Sport Assessment-Patella (VISA-P) score [20], which is a reliable and valid tool [21]. Every player received standardized information and a demonstration of the jumps.

All the players orally consented to participate in the study. According to the French Law, no written consent was needed due to the absence of modification of the routine players’ management. The data were anonymized before analysis. The local ethics committee (Comité Nantais d’Ethique en Médecine du Sport) approved the protocol under the number of registration CNEMS-2021-004. The study was also declared to the Research Department of the University Hospital. The study was in accordance with the Declaration of Helsinki [22].

### 2.2. Isokinetic Assessments

A Humac^®^ isokinetic dynamometer (Medimex, Sainte-Foy-lès-Lyon, France) was used to assess knee concentric isokinetic strength. This type of dynamometer uses a closed-loop control to respond to the movements and variations in the athlete’s peak of strength. It is based on four sub-systems: control–command, drive, mechanisms and measurement [23]. The control–command system is the operator–machine interface that sets each mode of exercises performed by the participants. The drive system is an electromechanical motor connected to the mechanism system through a reducer that provides resistance load. The driver is a power amplifier that manages and provides the voltage and electric current needed to the motor, using the reference in the control-command system. The mechanism system executes the patient–machine interface that allows the athlete to perform the evaluation in a comfortable posture, permitting the isolation of the work of the extensors or the flexors muscle group. The torque assessment is recorded by the measurement system from the electric motor current. Speed and position sensors are effective by encoder models. During the isokinetic evaluation, participants perform a muscular contraction at a predefined angular speed, constant during the range of motion except at the beginning (acceleration) and at the end (deceleration). The variation in velocity is managed by the computer’s pilot of the angular speed to prevent velocity overshoot [24]. The dynamometer provides resistance immediately in the proportion of the strength produced by the participant during the full range of motion of the joint.

The same Doctor of Physical Medicine and Rehabilitation conducted all the assessments in the conditions of use previously described [15,25,26]. Briefly, a 10-min ergocycle warm-up was realized before each session. A hip angle of 85° associated with the alignment of both the mechanical axis of the dynamometer and the lateral epicondyle of the knee was required for the starting position. Some belts stabilized the player’s trunk and pelvis. The knee range of motion was from full knee extension (0°) to a partial flexion (100°). Torque was gravity-corrected at 45° of knee flexion, and the dynamometer recalibration was performed monthly as requested by the manufacturer. In order to become familiar with the isokinetic movements, the players performed three submaximal and then two maximal movements.

For the assessment in concentric mode, one training period consisted of three repetitions at the angular speed of 60°/s and then followed by the second period of five repetitions at 180°/s, separated by a 30 s recovery period. Players were encouraged and received visual support.

We used the relative concentric isokinetic knee torques normalized to bodyweight. The knee extensors and flexors Limb Symmetry Index (LSI) was calculated to achieve a ratio below 1, allowing to be free for the dominant side [26]. In the PT group, the affected leg to the healthy leg formula was used, whereas, in the control group, the weak leg to the strong leg formula was applied.

### 2.3. Jumping Assessments

We used the conditions previously described by Dauty et al. to evaluate the jumps [26]. For the single-leg CMJ, an Abalakov belt was used with a precision of 1 cm. Each player realized a CMJ on one leg and then jumped vertically as high as possible with a landing on two feet [27]. They started with the leg of their choice, then alternated between both sides to achieve a total of three maximal jumps with each leg, all without using their arms. If the player continued to improve his jumping performance, additional trials could be realized until a plateau was reached in terms of height. For each leg, the relative distance, in relation to body weight, of the best jump was used for data analysis. The CMJ LSI was calculated with the same method as the isokinetic LSI.

For the one-leg hop test, a tape meter with a precision of 1 cm was used. Each player had to launch, land and balance on arrival for two seconds on the same foot, all without using his arms. As for the CMJ assessment, the relative distance, in relation to body weight, of the best jump was used for data analysis for each leg. The hop LSI was calculated as the isokinetic and the CMJ LSI.

### 2.4. Statistical Analysis

Data analyses were performed with SPSS 23.0^®^ software (Armonk, NY, USA). Quantitative parameters were presented as means and standard deviations, and qualitative parameters were expressed as absolute values. A first statistical analysis considered the volleyball players as units [28]. The normality of the tested parameters was assessed by a Kolmogorov–Smirnov test. A Levene test for homogeneity of variances was performed to compare quantitative data of volleyball player groups with and without PT, followed by a Student’s *t*-test. A link between the relative isokinetic strength and the relative jump performance was assessed with a Spearman test (*r*). The correlation coefficient was interpreted as strong correlation (*r* > 0.9), high (0.7 < *r* < 0.9), moderate (0.5 < *r* < 0.7), low (0.3 < *r* < 0.5), or negligible (*r* < 0.3) [29]. The qualitative data were compared through the use of a χ^2^ test, followed by a Bonferroni post hoc test. A second statistical analysis considered the lower limbs as units (“healthy legs”, “PT+ legs”, “PT− legs”) [28]. Due to the non-normal distribution of values, we performed a Kruskal–Wallis test followed by a Dunn post hoc test to compare these different groups, considering each lower limb independently. A significant difference was established for an α-level of 0.05.

## 3. Results

### 3.1. Participants

A total of thirty-six professional volleyball players were evaluated in this study. They all played in the French Premier League volleyball. They were 24.8 ± 5.2 years old, had a mean body weight of 88.0 ± 10.8 kg, and a mean height of 195.3 ± 8.1 cm. Eleven players were outside hitters, nine middle blockers, seven setters, five liberos and four opposite hitters. The prevalence of unilateral PT was 41.7% (15/36) (Table 1).

### 3.2. Comparison of the Control and PT Groups

The two groups were comparable in age, height and weight. The distribution of PT according to the position was different, especially for the setters and the liberos (*p* = 0.05). In the PT group, the VISA-P score was significantly lower than in the control group (78.7 ± 12.2 vs. 98.8 ± 2; *p* < 0.0001) (Table 1). No difference was found between the two groups in terms of isokinetic strength LSI or jump performance LSI (Table 2).

### 3.3. Comparison of the Lower Limbs as Units

The affected legs in the PT group (PT+) had a significant decrease in quadriceps strength at 60 and 180°/s compared with the healthy legs (*p* < 0.001). Hamstring strength also decreased at 60°/s (*p* < 0.001), but only a trend was found at 180°/s (*p* = 0.053). The hamstring/quadriceps strength (H/Q) ratio was significantly different both at 60 and 180°/s (*p* < 0.01 and *p* < 0.001, respectively) (Table 3).

The unaffected legs in the PT group (PT−) also had a significant decrease in quadriceps strength at 60 and 180°/s compared with the legs of the group without PT (*p* < 0.001). Hamstring strength was only decreased at 60°/s (*p* < 0.05), and there was only a difference in the H/Q ratio at 60°/s (*p* < 0.05) (Table 3).

No difference was identified between the strength of the PT+ legs and the strength of the PT− legs in the PT group (Table 3).

The PT+ limbs had a significant decrease in the relative CMJ and Hop test results compared with the lower limbs of the group without PT (*p* < 0.05 and *p* < 0.001, respectively). Despite lower values, the jump results were not significantly different between PT− limbs and healthy limbs. No difference was found between the PT+ and the PT− legs (Table 3).

### 3.4. Correlations between Isokinetic Strength and Jumping Performances

A low significant correlation was established between the relative isokinetic strength of the quadriceps and the relative CMJ value (*p* < 0.01). Likewise, a low relationship was found between the relative quadriceps isokinetic strength at 60°/s and the relative hop test value (*p* < 0.01). Concerning the correlation between hamstring strength and jumping performances, a negligible link was only present between the relative hamstring isokinetic strength at 180°/s and the relative CMJ value (*p* < 0.05). A moderate significant correlation existed between the two types of jumps evaluated in this study (Table 4).

## 4. Discussion

At the start of three professional volleyball seasons, we found a PT prevalence of 41.7%, leading to a low VISA-P score, a bilateral loss of knee strength and an impaired ability to jump in terms of CMJ and hop tests on the symptomatic legs. Nevertheless, the strength LSI and the jump LSI were comparable between the players with PT and those without PT.

Concerning the prevalence of PT in elite athletes, our results were in keeping with the literature, which reports a prevalence of 44.6% in volleyball [4]. In fact, volleyball is one of the most common sports responsible for PT due to the repetition of jumps during sports practice [19]. Consequently, a low VISA-P score was expected, as already shown in elite athletes from different sports with a score of 64 ± 19 [4]. Interestingly, it was demonstrated that significant correlations existed between pain, VISA-P score and isometric strength [30]. However, it is important to note that the majority of athletes continue to play despite the pain [31], underlining the importance of an objective assessment of the deficiencies such as isokinetic strength or jumping performances.

The impairment of isokinetic muscle strength remains unclear as only a few studies reported no significant difference in isokinetic quadriceps muscle strength when comparing a group with PT and a control group of runners or a mix of volleyball, basketball and handball players [13,32]. However, as previously published by Dauty et al. [26] in professional basketball players, our results support a significant decrease in the quadriceps strength in the case of PT. Concerning the hamstrings, we found a significant decrease in the strength at 60°/s and only a trend toward a difference at 180°/s, whereas other studies in basketball players and in runners showed no significant difference for this group of muscles [13,26]. This finding seems of great interest because it means that rehabilitation should also focus on hamstrings and not only on quadriceps in the case of PT.

Other methods were also used to study knee and hip strength in subjects with PT and found discordant results. For example, in isometric assessment, Scattone Silva et al. [31] showed a knee extensor torque comparable between the PT group and the control group, whereas Crossley et al. [14] demonstrated a decreased knee extensor torque. In three-dimensional motion analysis, Souza et al. [33] demonstrated a greater contribution from the hip extensors and a lower one from the knee extensors to perform a hop test. Conversely, Scattone Silva et al. [31] found a diminution in hip extensor torque in PT.

In contrast to our study, Lian et al. [18] showed, by using a composite jump score and a dynamic testing program of the leg extensors, a significant difference favorable to the PT group in volleyball players. They explained this result by the adapted training, which particularly targeted the strength of the extensor apparatus. In a similar way, Lian et al. [34] described an improvement in jumping performances in injured players, especially for CMJ. Another hypothesis for this phenomenon is that a player with a stronger knee extensor is more likely to develop PT. Moreover, several studies support the model that the excessive load leads to PT but also contributes to developing strength [11,35,36]. As a result, strength remains in the foreground even if PT develops. Likewise, in basketball players, no significant difference was found between PT groups and control groups in CMJ and the hop tests [26]. These findings were supported by the fact that players continued to compete despite knee pain associated with a low VISA-P score and that jumping ability depended on a complex set of muscles, not just on quadriceps or hamstring strength [26]. When considering these findings, our results are particularly interesting with regard to the decline in jumping performances in still active professional volleyball players, which is consistent with the results of Crossley et al. [14], who showed a lower knee extensor strength and a lower hop test in a unilateral PT group compared with a control group.

Indeed, the quadriceps Atherogenic Muscle Inhibition (AMI) is well known in the context of knee injuries [37]. AMI is usually responsible for a protective quadriceps inhibition to avoid over-utilization of the injured knee. It is due to the stimulation of joint receptors related to swelling, inflammation, laxity, or all types of articular damage. Supraspinal and spinal reflex pathways seem to be involved in pathogenicity [38]. As previously described, AMI can be present on the non-affected side in case of knee surgery, pain or arthropathy [38,39,40]. Our study suggested the presence of AMI in the extensor apparatus and, to a lesser extent, in the flexor apparatus of both legs in the PT group. This may be responsible for the lack of difference when comparing LSI between the PT and the control groups. Rio et al. [41] also showed that people with PT presented higher amounts of cortical quadriceps inhibition in the PT group than in the control group, so they proposed that their participants’ heavy isometric exercises contributed to a reduction in pain, which in return reduced cortical muscle inhibition [38,41]. Our findings on contralateral AMI could have a significant impact on PT management, particularly regarding strength recovery, a hypothesis supported by Rice et al. in 2010 [38,40]. Indeed, it was previously shown that contralateral rehabilitation had positive effects on ipsilateral strength in several limb injuries [42].

Concerning the correlation between CMJ and hop tests in players with PT, this result was expected as previously published in healthy male rugby players [43].

### Limitations

This study had several limitations. Firstly, only adult male volleyball players were included, preventing generalizing of the results to other populations. Secondly, the diagnosis was not completed by a radiological examination which could have helped eliminate differential diagnoses. However, no correlation between imaging such as ultrasound and clinical presentation was proven in this context of PT [5,44]. Thirdly, the method of evaluation, i.e., isokinetic, remains debatable given the plurality of possible types of contraction mode when performing jumps. However, isokinetic testing is considered a gold standard for knee strength evaluation due to its high reliability [45,46]. Finally, our study is limited by its cross-sectional design, as it prevented us from knowing exactly the mechanisms involved in PT appearance. A prospective follow-up would be necessary to clarify the results found, particularly the presence of AMI in the unaffected knee and its persistence over time.

## 5. Conclusions

Combing functional assessments were interesting to screen volleyball players with PT at the beginning of the sports season. Evaluating the players using the VISA-P score and isokinetic could highlight deficits related to PT. Indeed, volleyball players with PT showed an expected decrease in their VISA-P score but also a decrease in the isokinetic knee strength, both on the quadriceps and the hamstrings. Interestingly, this strength deficit was found both on the symptomatic legs and the asymptomatic ones. However, the relative values of CMJ and hop tests were only significantly altered on the pathological legs. Knowing the contralateral consequences in case of patellar tendinopathy may allow enhanced guidance of the volleyball players’ rehabilitation management in order to limit painful practice and loss of playing time.

## Figures and Tables

**Table 1:** Description and comparison of professional volleyball players according to the presence or the absence of patellar tendinopathy.

	All Volleyball Players (*n* = 36)	PT Group (*n* = 15)	Control Group (*n* = 21)	*p*
Age (years)	24.8 ± 5.2	26.5 ± 5.6	23.8 ± 4.8	0.13
Weight (Kg)	88.0 ± 10.8	91.5 ± 9.8	85.5 ± 11	0.10
Height (cm)	195.3 ± 8.1	197.8 ± 6.8	193.5 ± 8.6	0.12
Positions:				
Outside Hitters	11	4	7	0.05 ^$^
Middle Blockers	9	3	6	
Setters	7	6 ^$^	1	
Liberos	5	0 ^$^	5	
Opposite Hitters	4	2	2	
VISA-P score	90.4 ± 12.8	78.7 ± 12.2	98.8 ± 2	<0.0001

Abbreviations: PT: Patellar tendinopathy; BMI: Body Mass Index; VISA-P score: Victorian Institute of Sport Assessment—Patella score. ^$^ χ^2^ test followed by a Bonferroni test.

**Table 2:** LSI Comparison between volleyball players with and without patellar tendinopathy.

	Volleyball Players with PT (*n* = 15)	Volleyball Players without PT (*n* = 21)	*p*
Q60 LSI (%)	82.8 ± 11.6	89.1 ± 13.6	0.16
Q180 LSI (%)	90 ± 9.7	90 ± 8.6	0.96
H60 LSI (%)	93.4 ± 6.1	93.3 ± 6.9	0.97
H180 LSI (%)	93.4 ± 5.3	91.5 ± 5.1	0.29
CMJ LSI (%)	92.3 ± 7	91.4 ± 6.6	0.68
Hop LSI (%)	95.9 ± 5.1	95.4 ± 8.6	0.87

Abbreviations: PT: Patellar Tendinopathy; Q60 LSI: Quadriceps Limb Symmetric Index at 60° of isokinetic angular speed; H: Hamstring; CMJ: Countermovement Vertical Jump.

**Table 3:** Comparison between the affected legs with patellar tendinopathy (PT+ legs), the unaffected legs in patients with contralateral patellar tendinopathy (PT− legs) and the legs of control volleyball players (healthy legs) (Kruskal–Wallis test followed by Dunn post hoc test).

	PT+ Legs PT Group (*n* = 15)	PT− Legs PT Group (*n* = 15)	Healthy Legs Control Group (*n* = 42)	*p*
Q60 (Nm/kg)	2.14 ± 0.39 ^a^ ***	2.38 ± 0.33 ^b^ ***	3.04 ± 0.51 ^a^ ***^,b^ ***	<0.001
Q180 (Nm/kg)	1.62 ± 0.16 ^a^ ***	1.75 ± 0.27 ^b^ ***	2.09 ± 0.25 ^a^ ***^,b^ ***	<0.001
H60 (Nm/kg)	1.39 ± 0.20 ^a^ ***	1.47 ± 0.24 ^b^ *	1.62 ± 0.19 ^a^ ***^,b^ *	<0.001
H180 (Nm/kg)	1.16 ± 0.14	1.17 ± 0.21	1.27 ± 0.12	0.053
H/Q60	0.67 ± 0.15 ^a^ **	0.62 ± 0.10 ^b^ *	0.56 ± 0.12 ^a^ **^, b^ *	<0.01
H/Q180	0.72 ± 0.09 ^a^ ***	0.68 ± 0.11	0.61 ± 0.08 ^a^ ***	<0.001
Relative CMJ test (cm/kg)	0.44 ± 0.09 ^a^ *	0.46 ± 0.10	0.51 ± 0.09 ^a^ *	0.02
Relative hop test (cm/kg)	2.51 ± 0.32 ^a^ **	2.55 ± 0.32	2.80 ± 0.42 ^a^ **	0.004

Abbreviations: Q60 LSI: Quadriceps Limb Symmetric Index at 60° of isokinetic angular speed; H: Hamstring; CMJ: Countermovement Vertical Jump; ^a^ Significant difference between symptomatic PT+ limbs and controls; ^b^ Significant difference between asymptomatic limbs PT− and controls; Dunn’s test: * *p* ≤ 0.05; ** *p* ≤ 0.01; *** *p* ≤ 0.001.

**Table 4:** Spearman’s correlation between relative isokinetic strength and jump performances in volleyball players.

	Q60/kg	Q180/kg	H60/kg	H180/kg	Hop/kg	CMJ/kg
CMJ/kg	0.372 **	0.327 **	0.139	0.244 *	0.610 **	1
Hop/kg	0.376 **	0.127	0.010	−0.122	1	0.610 **

Abbreviations: Q60/Kg: Relative Quadriceps strength at 60 degrees per second per Kilogramme; H: Hamstring; CMJ/Kg: Relative Countermovement Jump per kilogramme; Hop/Kg: Relative Hop per kilogramme; PT: Patellar Tendinopathy. * *p* < 0.05; ** *p* < 0.01.

## Data Availability

The data presented in this study are available on request from the corresponding author. The data are not publicly available due to ethical reasons.
